# Crystal structures of GI.8 Boxer virus P dimers in complex with HBGAs, a novel evolutionary path selected by the Lewis epitope

**DOI:** 10.1007/s13238-014-0126-0

**Published:** 2014-12-31

**Authors:** Ning Hao, Yutao Chen, Ming Xia, Ming Tan, Wu Liu, Xiaotao Guan, Xi Jiang, Xuemei Li, Zihe Rao

**Affiliations:** 1 National Laboratory of Biomacromolecules, Institute of Biophysics, Chinese Academy of Sciences, Beijing, 100101 China; 2 University of Chinese Academy of Sciences, Beijing, 100049 China; 3 Division of Infectious Diseases, Cincinnati Children’s Hospital Medical Center, Cincinnati, OH 45229 USA; 4 University of Cincinnati College of Medicine, Cincinnati, OH 45267 USA

**Keywords:** norovirus, P domain, histo-blood group antigens (HBGAs), crystal structure, norovirus-host interaction

## Abstract

Human noroviruses (huNoVs) recognize histo-blood group antigens (HBGAs) as attachment factors, in which genogroup (G) I and GII huNoVs use distinct binding interfaces. The genetic and evolutionary relationships of GII huNoVs under selection by the host HBGAs have been well elucidated via a number of structural studies; however, such relationships among GI NoVs remain less clear due to the fact that the structures of HBGA-binding interfaces of only three GI NoVs with similar binding profiles are known. In this study the crystal structures of the P dimers of a Lewis-binding strain, the GI.8 Boxer virus (BV) that does not bind the A and H antigens, in complex with the Lewis b (Le^b^) and Le^y^ antigens, respectively, were determined and compared with those of the three previously known GI huNoVs, i.e. GI.1 Norwalk virus (NV), GI.2 FUV258 (FUV) and GI.7 TCH060 (TCH) that bind the A/H/Le antigens. The HBGA binding interface of BV is composed of a conserved central binding pocket (CBP) that interacts with the β-galactose of the precursor, and a well-developed Le epitope-binding site formed by five amino acids, including three consecutive residues from the long P-loop and one from the S-loop of the P1 subdomain, a feature that was not seen in the other GI NoVs. On the other hand, the H epitope/acetamido binding site observed in the other GI NoVs is greatly degenerated in BV. These data explain the evolutionary path of GI NoVs selected by the polymorphic human HBGAs. While the CBP is conserved, the regions surrounding the CBP are flexible, providing freedom for changes. The loss or degeneration of the H epitope/acetamido binding site and the reinforcement of the Le binding site of the GI.8 BV is a typical example of such change selected by the host Lewis epitope.

## INTRODUCTION

Human noroviruses (huNoVs), members of the *Norovirus* genus in the family *Caliciviridae*, are the most important viral pathogens of epidemic acute gastroenteritis in humans, causing significant morbidity and mortality worldwide. Noroviruses (NoVs) are non-enveloped RNA viruses covered by a protein capsid that encapsulates a single-stranded, positive-sense RNA genome of ~7.5 kb, encoding six functional and two structural proteins. NoVs are genetically diverse, comprising of six genogroups (GI to GVI) and over 35 genetic clusters or genotypes (Zheng et al., 2006), in which GI and GII constitute the majority of huNoVs. Structurally, NoV capsids exhibit a T = 3 icosahedron formed by 180 VP1s, the single major structure protein, organized into 90 dimers. NoV VP1 has two principle domains, the shell (S) and the protruding (P) domains, linked by a short, flexible hinge. The S domain builds the interior shell that forms the basic structure of the icosahedral capsid (Prasad et al., 1999), while the P domain dimerizes constituting the arch-like protrusions extending from the shell. The P dimer protrusions contain variable sequences and play an important role in virus-host interaction and immune responses of NoVs.

HuNoVs recognize histo-blood group antigens (HBGAs) as attachment factors or receptors which play an important role in the host susceptibility to huNoV infections. HBGAs are complex carbohydrates with specific oligosaccharide sequences as determinants of blood types, including A/B/O, secretor (H), and Lewis (Le) or non-secretor types (H negative). In addition to red blood cells, HBGAs distribute extensively on mucosal epithelia of intestinal tract, where they serve as attachment factors for huNoVs to initiate an infection (Tan and Jiang, 2014, 2011, 2010). HBGAs are also presented in saliva and mother milk, providing convenient reagents for *in vitro* study of huNoV-HBGA interactions. HuNoVs interact with HBGAs in strain-specific manners and complex interaction patterns between the diverse NoVs and the polymorphic HBGAs have been described (Huang et al., 2003, 2005). The role of human HBGAs in the host susceptibility or resistance to huNoV has been demonstrated by human challenge studies of both GI and GII NoVs (Frenck et al., 2012; Hutson et al., 2002; Lindesmith et al., 2003) and by investigations of outbreaks caused by NoVs (Nordgren et al., 2013; Tan et al., 2008).

HuNoVs are difficult to study due to the lack of a cell culture system and a small animal model. As a result, study of huNoV-host interactions relies on the recombinant virus-like particles (VLPs) and other subviral particles that self-assemble through expression of the VP1 and its subdomains. Earlier studies mapped the HBGA binding interfaces in the P domain (Tan et al., 2004, 2008) that formed P dimers (Tan et al., 2004) and/or P particles (Tan et al., 2008, 2011; Tan and Jiang, 2005), when the P domain is expressed in *E. coli*. X-ray crystallography of NoV P dimers in complex with HBGA oligosaccharides further elucidated the structures of the HBGA-binding interfaces in atomic resolution (Bu et al., 2008; Cao et al., 2007; Chen et al., 2011; Choi et al., 2008; Hansman et al., 2011; Kubota et al., 2012; Shanker et al., 2011). The conformational HBGA binding interfaces, formed by several scattered amino acids of the P domain, are located on the top of the arch-shaped P dimers, corresponding to the outermost surface of the viral capsid. Interaction networks between the terminal saccharides of HBGAs and the amino acids forming the binding interfaces have been thoroughly described [reviewed in (Tan and Jiang, 2014, 2011, 2010)]. While the locations and the major amino acids that form the core structure of the HBGA binding interfaces are apparently conserved among different genotypes within GI and GII NoVs, the two genogroups use completely different HBGA binding sites in interacting with the same repertoire of human HBGAs, indicating distinct evolution paths of the two genogroups of huNoVs.

The structural basis of GII NoV-HBGA interactions has been thoroughly elucidated through studies of the HBGA-binding interfaces of five GII NoVs, representing different genetic backgrounds (GII.4 1997 variant, GII.4 2004 variant, GII.9, GII.10 and GII.12) and various HBGA binding profiles (A, B, H, Le and nonsecretor) (Cao et al., 2007; Chen et al., 2011; Choi et al., 2008; Hansman et al., 2011; Shanker et al., 2011). All these GII NoVs interact with variable HBGAs through a conserved binding interface. A highly conserved fucose (Fuc)-binding pocket at the center of the binding interface is identified as central binding pocket (CBP) that plays a major role in interaction with HBGAs via the α-1,2 Fuc or α-1,3/4 Fuc as the major binding saccharides (MaBSs) (Tan and Jiang, 2014). In addition, one or two other adjacent saccharides also participate in binding to the surrounding regions of the CBP as minor binding saccharides (MiBS) to strengthen the binding forces. Thus, a successful binding of a GII NoV with an HBGA requires synergetic actions of both the CBP and the surrounding region with the MaBS and MiBSs, respectively. The relative conservation of the CBP and the variation in the surrounding region explained the genetic relatedness or evolutionary paths among NoVs within a genogroup selected by the polymorphic human HBGAs (Tan and Jiang, 2014).

**Figure 1:**
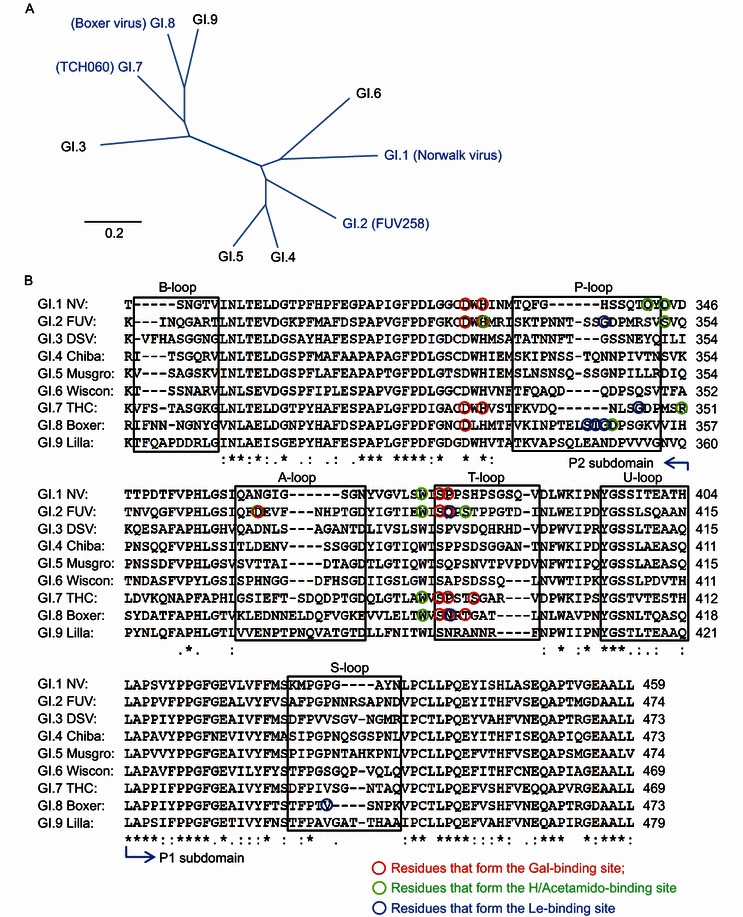
**Phylogeny of Boxer virus among GI NoVs and sequence alignment analyses**. (A) Phylogenic tree of the nine known GI genotypes with indications of Norwalk virus (GI.1), FUV258 (GI.2), TCH060 (GI.7), and Boxer virus (GI.8) whose crystal structures of the HBGA-binding interfaces have been determined. (B) Sequence alignments of partial P domains of representatives of the nine known GI genotypes. GI.1 NV, the prototype Norwalk virus (M87661); GI.2 FUV (BAC05516); GI.3 DSV, Desert Shield virus (AAA16285); GI.4 Chiba, Chiba virus (BAB18267); GI.5 Musgro, Musgrove virus (AJ277614); GI.6 Wiscon, Wisconsin virus (AY502008); GI.7 TCH, TCH060 (AEQ77282); GI.8 Boxer, Boxer virus (AF538679); and GI.9 Lilla, Lilla Edet virus (AEY77023). The five surface loops (A-, B-, P-, S-, and T-loops) and U-loop are shown by rectangular frames. The residues that form the galactose-, H epitope/acetamido- and Le epitope-binding sites of the HBGA binding interface are indicated by red, green and blue circles, respectively

However, our understanding of GI NoV-HBGA interactions remains limited due to the fact that the HBGA binding interfaces of only three GI NoVs, the Norwalk virus (NV, GI.1), FUV258 (FUV, GI.2) and TCH060 (TCH, GI.7) are known and the three NoVs revealed similar HBGA binding profiles to A/H/Le antigen (Bu et al., 2008; Choi et al., 2008; Kubota et al., 2012; Shanker et al., 2014), although NV binds only secretor (Le^b/y^) but not nonsecretor (Le^a/x^) Le antigen. However, GI NoVs are diverse in recognizing different HBGAs. The available structural data suggested that the galatose (Gal) binding site that interact with the β-1,3 Gal of the precursor or the α-1,3 Gal of the A-epitope, plays a central role in binding to various HBGAs (Bu et al., 2008; Choi et al., 2008), while the α-1,2 Fuc (H epitope) and α-1,3/4 Fuc (Le epitope) binding sites also play roles in binding to H and Le antigens (Bu et al., 2008; Choi et al., 2008; Kubota et al., 2012; Shanker et al., 2014). It remains elusive whether the H epitope/acetamido binding site is required for the Le binding strains that do not bind the H antigen. To this end, we selected Boxer virus (BV), a GI.8 clinical isolate, for further structural study. Genetically, GI.8, together with GI.9, constitutes a branch in the GI NoV phylogenetic tree (Fig. 1A) (Kroneman et al., 2013) and has the longest A- and P-, but shortest T-loops (Fig. 1B) that are heavily involved in the formation of the GI HBGA binding interfaces (Bu et al., 2008; Choi et al., 2008; Kubota et al., 2012; Shanker et al., 2014). Unlike NV, FUV and TCH that bind all A, H and Le antigens (NV binds A/H/Le^b/y^), BV binds only Le, but not A and H antigens (Huang et al., 2005). Thus, BV represents a unique model to further understand GI NoV-HBGA interactions. Our results showed that, corresponding to its binding profile, BV has a well-developed Le epitope-binding site formed by five amino acids, being the largest Le epitope binding site of GI NoVs. On the other hand, the H epitope/acetamido-binding site observed in the other three GI NoVs is missing, explaining the inability of BV binding to A and H antigens. Our data emphasize the complexity of NoV-HBGA interactions and highlight the role of human HBGAs in the evolution of HBGA binding interfaces of NoVs.

## RESULTS

### Production, characterization and crystallization of BV P protein

Previous results showed that the GI.8 BV, a clinical isolate, bound uniquely to the Le, but not the A and H antigens (Huang et al., 2005), making the BV distinct from other tested GI NoVs that bind generally the A and H antigens (Shirato et al., 2008). Further sequence analysis revealed that GI.8, together with GI.9, forms a branch in the GI phylogenetic tree (Fig. 1A) (Kroneman et al., 2013) that has the longest A-, B- and P-, but the shortest S- and T-loops among the nine genotypes of GI NoVs (Fig. 1B). These different surface loops may be translated into distinct surface topology of BV compared with other GI NoVs and the P- and T-loops have been known to be heavily involved in the formation of the GI HBGA binding interfaces (Bu et al., 2008; Choi et al., 2008; Kubota et al., 2012; Shanker et al., 2014).

The BV P domain was first expressed as P particles (Tan and Jiang, 2005; Tan et al., 2009) to confirm its HBGA binding pattern. This revealed a typical BV binding profile to Le antigens, including the secretor Le^b^ and Le^y^, as well as the nonsecretor Le^a^, but not to A and H antigens (Huang et al., 2005). P dimer was then produced in *E. coli* at a yield of ~5 mg per liter culture. The purified and concentrated P dimer protein (>95% purity, 10 mg/mL) was crystallized in native form and in complex with Le^b^ and Le^y^ tetrasaccharides. The resulting crystals could diffract to high resolution beyond 1.6 angstrom (Å). The diffraction data were collected at synchrotron radiation centers and the structures were solved by molecular replacement method.

### The crystal structure of the native BV P domain

**Figure 2:**
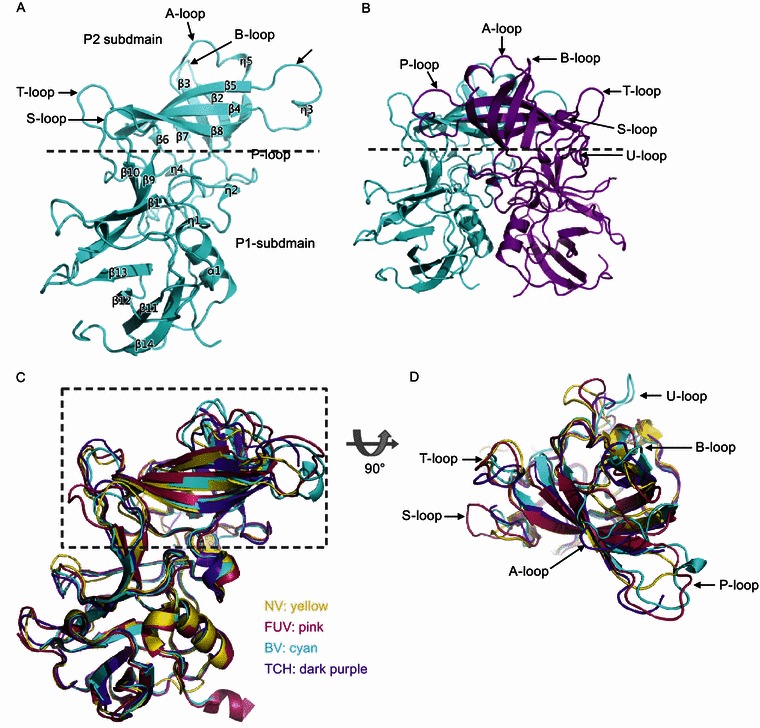
**Structures of Boxer virus (BV) P domain and its comparison with those of other GI P domains**. (A) Structures of the BV P domain monomer (ribbon model). (B) Structures of the BV P domain dimer (ribbon model). The dashed lines show the boundary between the P2 (up region) and the P1 (down region) subdomains. (C) Superimposes of the BV P domain monomer (BV, GI.8, cyan) with those of Norwalk virus (NV, GI.1, yellow), FUV258 (FUV, GI.2, pink) and TCH060 (TCH, GI.7, dark purple) (all ribbon models). (D) An enlargement of the top region of the superimpose [blue dashed rectangular region in (C)] of the P domains. The six surface loops (A-, B-, P-, S, T- and U-loops) are indicated

The native BV P protein was crystallized under the space group of P6_1_, containing two P protein protomers in an asymmetric unit, related by a non-crystallographic 2-fold axis. Peptide chain ranging from K230 to L526 (Fig. 2A) can be modeled, while distal parts of two loop regions in the P1 subdomain (see below) comprising Q260/N261 and G502/G503, respectively, could not be modeled due to un-interpretable local electron density maps. These regions still couldn’t be modeled in Le^b^ or Le^y^ complex structures, indicating internal flexibility properties of these regions.

BV P domain retains the basic structures of the GI NoV P domains (Fig. 2) (Bu et al., 2008; Choi et al., 2008; Kubota et al., 2012; Shanker et al., 2014). The P1 subdomain, constituted by the N′ (K230–A279) and C′ (A420–L526) terminal regions, forms mainly the legs of the arch-like P dimer (see below), while the P2 subdomain, formed by a large insertion from R280 to L419, builds the majority of the head of the P dimer (Fig. 2B). The P1 subdomain is a mixed α/β structure containing an amphiphile α helix and two twisted antiparallel β sheets: β1-β9-β10 and β9-β11-β12-β13-β14, where β9 strand was shared between the two β sheets (Fig. 2A). The P2 subdomain is constituted mainly by a β-barrel structure formed by two twisted antiparallel β sheets: β5-β4-β8 and β8-β2-β3-β7-β6. Superimposing BV P domain with the three GI homologs, GI.1 NV (Bu et al., 2008; Choi et al., 2008), GI.2 FUV (Kubota et al., 2012) and GI.7 TCH (Shanker et al., 2014) reveals that the P1 subdomain is very conserved among the four structures (r.m.s.d. = 0.74 Å compared with NV Cα atoms, r.m.s.d. = 0.71 Å compared with FUV, and r.m.s.d. = 0.95 Å compared with TCH) (Fig. 2C).

**Figure 3:**
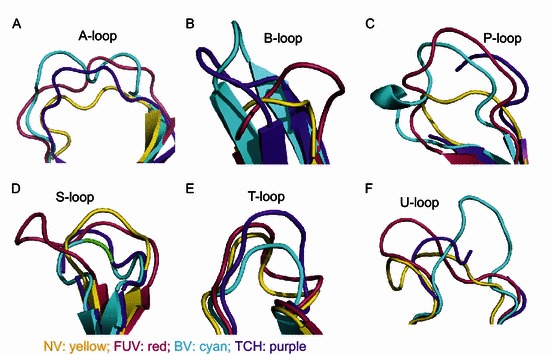
**Comparisons of the structures of the six surface loops of Boxer virus (BV, GI.8, cyan) individually with those of Norwalk virus (NV, GI.1, yellow), FUV258 (FUV, GI.2, pink) and TCH060 (TCH, GI.7, dark purple) (all ribbon models) through superimpose approach**. (A) A-loop; (B) B-loop; (C) P-loop; (D) S-loop; (E) T-loop; (F) U-loop

The major structural differences among the four P domains reside on 6 loop regions (Figs. 2D and 3), including the previously identified A-, B-, P-, T- and U-loops in the P2 subdomain and the S-loop in the P1 subdomain (Kubota et al., 2012; Shanker et al., 2014), consistent with the variations, insertions and/or deletions of the sequences of the loops (Fig. 1). It was noted that, although the U-loop shares the same length, it shows striking structural differences among the four strains, indicating both length and sequences determine the structures of the loops. These variable loops have conferred BV unique surface topology differing from the other three GI NoVs (see below).

### The structure of the BV P dimer

**Figure 4:**
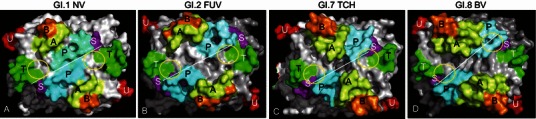
**Comparisons of the surface structures of the six loops among the P dimers (surface models) of Norwalk virus (GI.1 NV), FUV258 (GI.2 FUV), TCH060 (GI.7 TCH) and Boxer virus (GI.8 BV)**. The A-, B-, P-, S-, T- and U-loop regions (surface model) are indicated by different colors. The boundaries between the two P monomers are shown by dashed lines. The locations of the HBGA binding interfaces are labeled by dashed circles

As expected, the P protein is dimerized via a non-crystallographic 2-fold axis, forming an arch-like P dimer (Fig. 2B) in dimension of 54 Å × 62 Å × 67 Å. The P dimer is stabilized by the vast monomer buried surface area of 3,733 Å^2^ (including two monomers) comprised of both hydrophobic and hydrophilic residue interactions contributed from both P1 and P2 subdomains. The five loops (A-, B-, P- S- and T-loops, Fig. 1) occupy the majority of the top surface of the BV P dimer (Fig. 3). Corresponding to their maximum lengths, both A- and B-loops of BV are more exposed than those of GI.1 NV, GI.2 FUV and GI.7 TCH (Figs. 3 and 4). In addition, the two P-loops of a P dimer that are the major components of the Le epitope binding sites (see below) are closer to each other in GI.1 NV, occupying the central area of the top surface of the P dimer. They move sideward in the other three GI NoVs (FUV, TCH and BV) forming the Le epitope binding sites, which reach to maximum extension to the HBGA binding site in BV (Fig. 4) (Kubota et al., 2012; Shanker et al., 2014). In contrast, the S- and T-loops become shorter and less exposed in BV compared with other three GI counterparts. Therefore, the five loops with different lengths and sequence variations change heavily the surface topologies of the P dimers, which may be further translated into different antigenic features of the GI NoVs.

### The HBGA binding interface of BV

**Figure 5:**
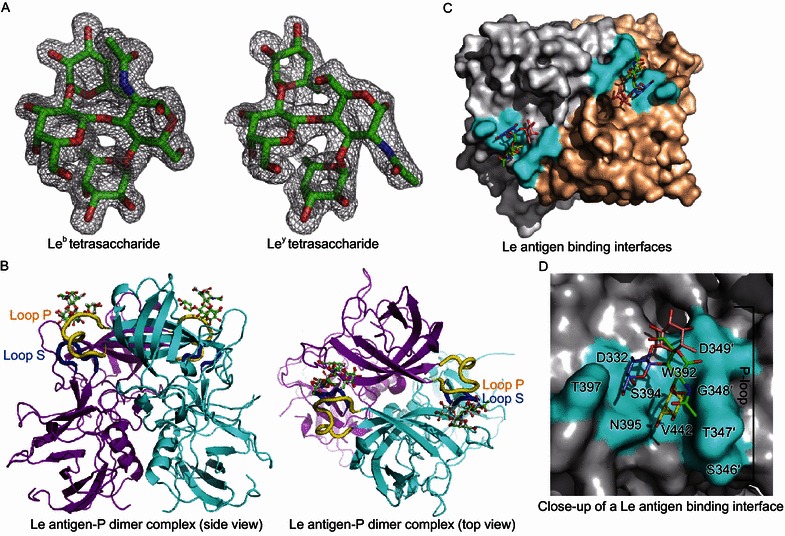
**Structures of Boxer virus (BV) P dimers in complex with the Le**^**b**^**and Le**^**y**^**tetrasaccharides**. (A) Fourier difference maps of the Le^b^ (left) and Le^y^ (right) tetrasaccharides. The electron density maps were calculated from diffraction intensity F_complex_− F_native_ with phase angle of native protein structure contoured at 1.6 *σ* (grey). (B) Structures of a BV P dimer (ribbon model) with Le^b^ tetrasaccharides (stick and ball model) in side (left) and top (right) views, respectively. The two P monomers are shown in purple and cyan, respectively, while the P- and S-loops that are involved in the formation of the Le epitope binding site are in yellow and blue. (C) Top view of the BV P dimer (surface model) with indications of the two P monomers (grey and sand, respectively), Le^b^ tetrasaccharide (stick model), and the amino acids forming the HBGA binding interface (cyan). (D) A close-up of the Le antigen binding interface with indication of each amino acid component. Numbers with prime (′) indicate residues of the other P protomer. In (C) and (D) the amino acids forming the HBGA binding interface are in cyan. The individual saccharides of the Le^b/y^ tetrasaccharides are shown in different colors: H epitope, pink; Gal, purple; GlcNAc, green; Le epitope, yellow. The P-loop is indicated

The BV P dimers in complex with type 1 Le^b^ and type 2 Le^y^ tetrasaccharides, respectively, were crystallized in the same space group and unit cell dimension of native protein (Table 1) and thus may be viewed as isomorphous with the native crystal. Fourier difference maps were calculated from diffraction intensity F_complex_ − F_native_ combined with phase angle derived from final native protein structure, which unambiguously reveals the high resolution electron density maps of the bound tetrasaccharides (Fig. 5A). The structure of tetrasaccharides were modeled and optimized by the guidance of electron density maps and stereo-chemical restraints. Statistics of the final optimized complex structures are summarized in Table 2 and torsion angles φ and ψ of the final tetrasaccharides are included in Table 3.

**Table 1:** Data collection statistics

Parameters		Native P protein	Complex with Le^b^ tetrasaccharide	Complex with Le^y^ tetrasacharide
Spacegroup		*P6*_*1*_	*P6*_*1*_	*P6*_*1*_
Resolution range^a^		50–1.50 (1.53–1.50)	50–1.63 (1.66–1.63)	50–1.45 (1.48–1.45)
Cell dimensions (Å)	*a*	139.9	139.9	140.4
	*b*	139.9	139.9	140.4
	*c*	64.7	64.8	65.0
Total no. of reflections		1,289,366	896,187	1,433,231
No. of unique reflections		115,571	90,264	129,481
Completeness (%)^a^		100.0 (100.0)	100.0 (100.0)	100.0 (100.0)
Redundancy^a^		11.2 (11.0)	9.9 (8.1)	11.1 (11.0)
*I*/*σ*(*I*)^a^		42.9 (5.4)	43.2 (3.6)	27.7 (6.1)
*R*_merge_ (%)^a,b^		6.1 (45.8)	7.9 (50.3)	9.5 (47.3)

^a^ Values in parentheses correspond to the shell of highest resolution

^b^*R*_merge_ = ∑_hkl_| *I*_i_ − *I*_m_ | / ∑_hkl_ < *I*_m_ > , where *I*_i_ and *I*_m_ are the observed and mean intensity of related reflections with common indices h, k, and l

**Table 2:** Structure refinement statistics

Parameters	Native P protein	Complex with Le^b^ tetrasaccharide	Complex with Le^y^ tetrasacharide
No. of reflections in working set	109,591	85,705	122,915
No. of reflections in test set	5,825	4,527	6,496
*R*_work_^a^	0.136	0.131	0.128
*R*_free_^a^	0.163	0.167	0.150
Root mean square deviation			
Bond lengths (Å)	0.006	0.006	0.009
Bond angles (º)	1.057	1.054	1.199
Average B factors (Å^2^)			
Total	19.9	27.1	20.1
Protein	17.8	25.3	17.5
Tetrasaccharide	–	28.5	26.8
Solvent	32.1	39.1	33.7
Residues in the ramachandran plot (%)			
Favored	97.9	97.4	97.4
Allowed	2.1	2.6	2.6
Disallowed	0.0	0.0	0.0

^a^*R*_work_ = ∑| |*F*_obs_| − |*F*_cal_| |/∑|*F*_obs_|, *R*_free_ = ∑_T_| |*F*_obs_| − |*F*_cal_| |/∑_T_|*F*_obs_|, where *F*_obs_ and *F*_cal_ are observed and calculated structure factors, respectively. For *R*_free_, T is a randomly selected test data set (5.0%) of total reflections and was set aside before structure refinement

**Table 3:** Torsion angles of glycosidic linkages in Le^b^ and Le^y^

Tetrasaccharides	Saccharides	phi(Φ)	Psi(Ψ)
Le^b^	α-1,4 Fuc	−70.5 (−75.2)	140.4 (141.0)
	β-1,3 Gal	−63.7 (−63.1)	−104.9 (−104.9)
	α-1,2 Fuc	−73.9 (−74.4)	129.2 (131.5)
Le^y^	α-1,3 Fuc	−71.6 (−76.6)	−93.7 (−99.0)
	β-1,4 Gal	−75.8 (−69.6)	129.7 (127.2)
	α-1,2 Fuc	−74.2 (−72.2)	122.9 (128.1)

^a^Values in parentheses correspond to the tetrasaccharide at the other binding pocket related by a non-crystallographic two fold axis in the dimer structure

**Figure 6:**
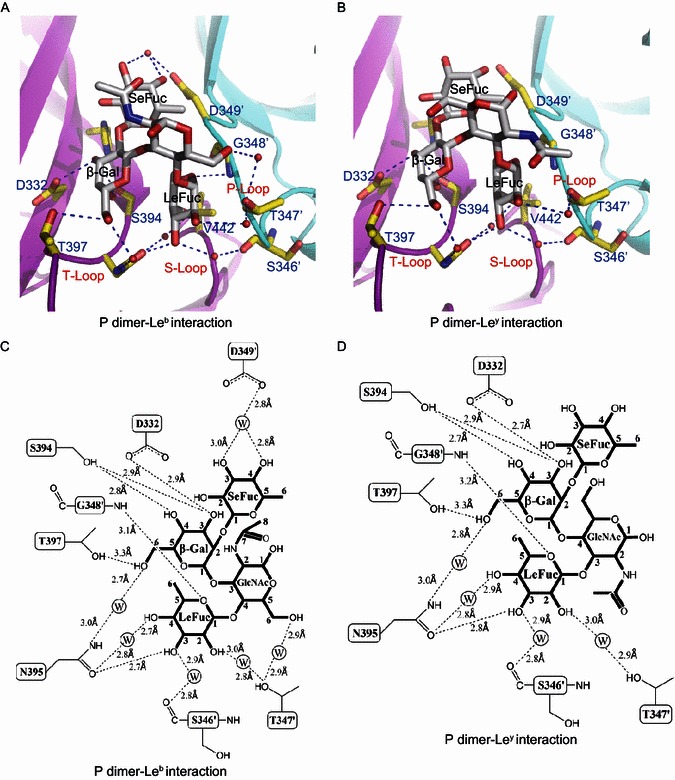
**The interaction networks between the HBGA binding interface of boxer virus (BV) and the Le antigens**. (A and B) The Le antigen binding interface of BV in complex with Le^b^ (A) and Le^y^ (B) tetrasaccharides, respectively, with indication of hydrogen bonds (dashed lines). The P dimer is shown in ribbon model in cyan and purple with indications of the amino acids (in stick model, yellow) that interact with the Le antigens. The P-, S- and T-loops are indicated by red fonts. The Le^b^ (A) and Le^y^ (B) antigens are shown in stick (grey) with indications of the β-1,3 galactose (β-Gal), the Le epitope (α-1,3/4 fucose, LeFuc), H epitope (α-1,2 fucose, SeFuc) and N-acetyle glucoseamine (GlcNAc). (C and D) Schematic illustrations of the detail hydrogen bond (dashed lines) network between amino acids of the binding interface and the individual saccharides of the Le^b^ (C) and Le^y^ (D) tetrasaccharides

**Figure 7:**
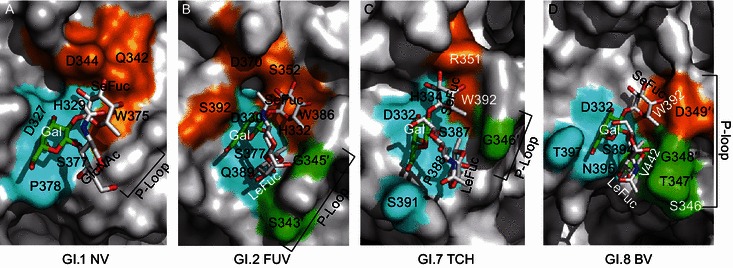
**Evolutionary changes of the topologies of the HBGA binding interfaces among GI.1 Norwalk virus, GI.2 FUV, GI.7 TCH and GI.8 Boxer virus (BV).** (A to D) Top views of the HBGA binding interfaces (surface models) of Norwalk virus (NV) (A), GI.2 FUV (B), GI.7 TCH (C) and GI.8 BV (D) with indications of the galactose (cyan), the H epitope/acetamido (orange) and the Le epitope (green) binding sites. The HBGAs are shown in stick with indications of the β-1,3 galactose (β-Gal, green), the Le epitope (α-1,3/4 fucose, LeFuc) (grey), H epitope (α-1,2 fucose, SeFuc) (grey) and N-acetyle glucoseamine (GlcNAc) (grey). Residues that form each of the HBGA binding sites are indicated. The locations of the P-loops are shown

Two symmetric HBGA binding interfaces are identified on the top of the BV P dimer at the common boundary between the two P monomers (Fig. 5), sharing similar locations with the other three known GI binding interfaces (Bu et al., 2008; Choi et al., 2008; Kubota et al., 2012; Shanker et al., 2014) (Fig. 7). The conformational binding interface of BV is composed of two major areas, a β-Gal binding site and a Le epitope binding site, formed by ten amino acids from 3 (P-, T-, and S-) loop regions (Fig. 5). The residues D332, S394 and V442 form the bottom of the pocket-like binding interface, while T397 and N395 forms a “wall”, and S346′, T347′, G348′ and D349′ of the P-loop from the other protomer formed the other “wall” of the pocket (Figs. 6 and 7). Noteworthy, only two (D332 and S394) of these amino acids are conserved among GI NoVs (Fig. 1B) and both residues are components of the β-Gal binding site (see below). BV binds the Le antigens mainly through two saccharides. The β-Gal of the precursor makes a major contact with the “bottom” of the binding interface, which is stabilized by residue T397, while the Le epitope, the α-1,3/4 Fuc (Le Fuc), contacts with another part of the “bottom” and is stabilized by the other “wall” (S346′, T347′, G348′ and D349′) to support the binding outcomes (Figs. 5D, 6 and 7D). Extensive hydrogen (H) bond networks are seen between the two saccharides and the amino acid residues of the binding interface, in which some interactions are mediated by water molecules (Fig. 6).

### The β-Gal binding sites

This is formed by four residues from a single P monomer, including two highly conserved (D332 and S394) and two variable (N395 and T397) residues (Figs. 1, 5D and 6). It interacts with the β-Gal of the precursor disaccharides through five H bonds, including a water-bridged hydrogen bond. This β-Gal binding site is conserved among all BV, NV, FUV and TCH with known crystal structures (Bu et al., 2008; Choi et al., 2008; Kubota et al., 2012; Shanker et al., 2014) and thus may be defined as the central binding pocket (CBP) of GI HBGA binding interfaces, because it plays the central role in GI NoV-HBGA interactions, as proposed previously (Tan and Jiang, 2014). The conservation of the β-Gal binding site may be a result of an evolutionary selection by HBGAs, as a prerequisite for viral survival.

### The Le epitope binding site

This is the other major area of the BV HBGA binding interface. It is formed by five variable residues from both P protomers, including three consecutive amino acids (S346′, T347′ and G348′) from the P-loop that extends from the other protomer (symbol ′ indicates residue of the opposite P protomer), V442 from the S-loop extending from the P1 subdomain and N395 that also interact with the β-Gal (Figs. 1, 5, 6 and 7). This site interacts with the Le epitope through five H bonds, including three water bridged H bonds. Thus, unlike GI.1 NV that lacks a Le epitope binding site and GI.2 FUV/GI.7 TCH that has a Le epitope binding site formed by one (FUV) or two (TCH) residues (Bu et al., 2008; Choi et al., 2008; Kubota et al., 2012; Shanker et al., 2014). Noteworthy, BV has a much larger, well-developed Le epitope binding site. These findings explain the binding capability of GI NoVs to Le antigens and highlight the role of HBGAs as a selection factor in the evolution of the NoV binding interfaces.

### The H epitope/acetamido binding site

Unlike the other three GI NoVs (NV, FUV and TCH) that the H epitope, the α-1,2 Fuc (SeFuc), plays an important role in interaction with the secretor HBGAs (Bu et al., 2008; Choi et al., 2008; Kubota et al., 2012; Shanker et al., 2014), this saccharide does not play a major role in BV-HBGA interaction (Fig. 6). The H epitope binding sites of NV, FUV and TCH are formed by two to five amino acids and none of these residues interacts with the H epitope in BV (Figs. 6 and 7), except for W392 that may form a hydrophobic interaction with the 6 methyl group of the H epitope. In fact, BV does not form a single H bond with the H epitope in binding to the type 2 Le^y^ antigen, while only one water mediated H epitope between D349′ of the P-loop and the H epitope was seen in BV binding to the type 1 Le^b^ antigen (Fig. 6). Therefore, the H epitope binding site observed in the other three GI NoV is missing or greatly degenerated in BV. Since the H epitope binding site also interacts with the acetamido group when the other three GI NoVs bind the A antigens, the loss of the H epitope/acetamido binding site explains the lack of binding of BV to the A and H antigens. Finally, BV also forms a water mediated H bond with the N-acetyl glucosamine (GlcNAc) of the precursor through T347′ of the P-loop in binding to the Le^b^ antigen, a scenario that has not yet been observed in the other three GI NoVs. Above comparisons of the BV binding interface with the other three GI NoV emphasize the conservation of the CBP and the variations of the other binding sites, two features that may confer NoVs both viability and adaptability (see Discussion).

### Validation of amino acids forming the binding interface by mutagenesis study

**Figure 8:**
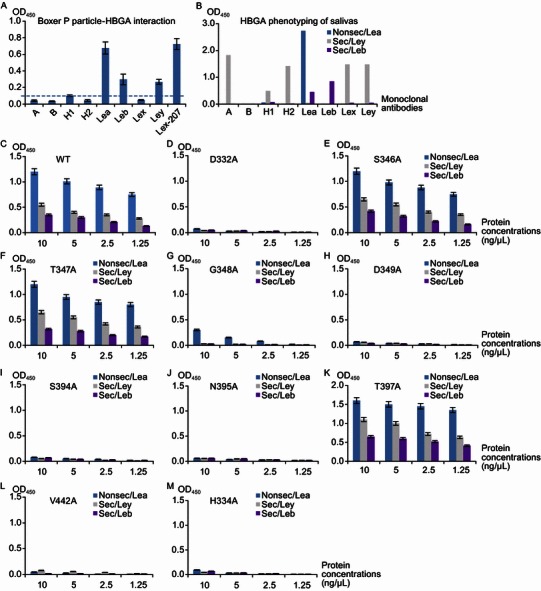
**HBGA-binding outcomes of wild type and various mutant P particles with single amino acid mutations in the HBGA-binding interface of Boxer virus (BV)**. (A) Binding of wild type BV P particles with a panel of oligosaccharides representing different HBGAs (A, B H1, H2, Le^a^, Le^b^, Le^x^ and Le^y^). The positive binding of VA207 P particles with Le^x^ antigen (Le^x^-207) serves as a positive control of Le^x^ antigen. VA207 (GII.9) is another Lewis binding NoV that has been shown to bind Le^x^ antigen. (B) HBGA phenotyping of saliva samples. The three saliva samples that were used for binding with mutant P particles (C–M) were typed phenotypically by various monoclonal antibodies specific to A, B H1, H2, Le^a^, Le^b^, Le^x^ and Le^y^ antigens. The concentrations of the P particles were 10 mg/mL. (C–M) HBGA-binding outcomes of wild type (C) and various mutant P particles with single amino acid mutations in the HBGA-binding interface of BV (D–M) with the well-defined saliva samples. The three saliva samples were positive with nonsecretor Le^a^ (Nonsec/Le^a^), secretor Le^y^ (Sec/Le^y^) and secretor Le^b^ (Sec/Le^b^), respectively. Y axes indicate the optical densities at 450 nm (OD450), while the X-axes indicate the concentrations of the P particles (ng/µL)

The wild type BV is known to bind Le^a^, Le^b^ and Le^y^ antigens (Fig. 8A and 8C). Binding changes of the individual mutants were determined using three saliva samples that are Le^a^ (Nonsec/Le^a^), Le^y^ (Sec/Le^y^) and Le^b^ (Sec/Le^b^) positive, respectively (Fig. 8B). As expected, most single mutations (D332A, G348A, D349A, S394A, N395A and V442A) lead to complete or nearly complete loss of binding (Fig. 8D, 8I–J and 8L), indicating that these residues are strictly required for the structural and functional integrity of the HBGA binding interface. Two mutants (S346A and T347A, Fig. 8E and 8F) retained the same binding activity, probably because: 1) the interacting atom oxygen (= O) of the carboxyl group of S346 is also present in the alanine; and 2) serine (S346), threonine (T347), and alanine are tiny amino acids, sharing similar structures, although how the mutated alanine replaces the function of the T347 and S346 remains to be defined. Interestingly, mutant T397A showed increased binding activity compared with that of the wild type (Fig. 8K), probably resulting from the removal of certain structure constraints by replacing the bigger “wall” (T397) with a smaller one (alanine) (see Discussion).

In addition, H334 residue that is structurally nearby the binding pocket was also examined. H334 is a conserved residue among GI NoVs that is involved in the formation of the Gal binding site of the other three GI NoVs. In BV it forms an H bond (~ 2.8 Å) with S394 to stabilize the conformation of the CBP. As expected, H334A mutation wiped out the binding of the BV (Fig. 8M). Thus, mutations at residues near the binding interface can also abolish the function of the binding interface. Noteworthy, changes of binding patterns through mutations of amino acids in the HBGA binding interfaces, a phenomenon that was often seen previously (de Rougemont et al., 2011; Kubota et al., 2012; Tan et al., 2008, 2009), does not occur among these mutations.

## DISCUSSION

Before our study, the crystal structures of the HBGA binding interfaces of three GI NoVs, the GI.1 NV, the GI.2 FUV and the GI.7 TCH, have been determined (Bu et al., 2008; Choi et al., 2008; Kubota et al., 2012; Shanker et al., 2014), providing valuable information for basic understanding of GI NoV-HBGA interactions. However, while these three GI NoVs represent different genetic types, they exhibit similar HBGA binding profiles to the A, H and Le antigens (Huang et al., 2005; Kubota et al., 2012), with only difference in binding to the non-secretor Le antigens (seLe, Le^a/x^). Indeed, in spite of great differences on the surface topology due to their sequence variations of the P domains, the three GI NoVs share similar structural features of the HBGA binding interfaces, including a Gal- and an H- epitope/acetamido binding site (Bu et al., 2008; Choi et al., 2008; Kubota et al., 2012; Shanker et al., 2014). These two binding sites confer the three GI NoVs a common binding capability to the A, H and secretor Le (SeLe, Le^b/y^) antigens. In addition, corresponding to the ability of FUV and TCH binding to the seLe antigens, these two GI NoVs develop an extra Le epitope binding site, making their binding interfaces trivalent, interacting with the Gal, H epitope/acetamido and Le epitope, respectively, while the NV binding interface is bivalent, consisting of the Gal and the H epitope/acetamido binding sites only.

While bindings to all A, H and Le antigens appear to be a general feature of GI NoVs (Shirato et al., 2008), different HBGA binding profiles of GI NoVs have also been observed (Huang et al., 2005). For example, the GI.8 BV represents a typical example of such special HBGA binding profiles. BV was isolated from a NoV gastroenteritis outbreak occurred on a battle ship of US Navy in 2001. Previous study showed that BV VLPs bound to the Le antigens only, including the SeLe and seLe antigens, but not to the A and the H antigens. In addition, GI.8, together with the GI.9 NoVs constitute a genetic branch that has the longest A-, B- and P-loops, but the shortest S- and T-loops among GI NoVs (Fig. 1). Earlier studies showed that the A-, P- and T-loops are involved in the formation of the HBGA binding interfaces (Bu et al., 2008; Choi et al., 2008; Kubota et al., 2012; Shanker et al., 2014). Thus, the different lengths of the surface loops of BV could be the major factors leading to the unique HBGA binding profile of BV.

In this study, the crystal structures of the BV P dimers in complex with the type 1 Le^b^ and the type 2 Le^y^ antigens, respectively, were resolved. The data showed that BV has a unique HBGA binding interface consisting of two major saccharide binding sites, each interacting with the β-1,3 Gal and the Le epitope. The β-1,3 Gal binding site is conserved with the other three GI NoVs, while the Le epitope binding sites are particularly well developed, formed by five residues and interact with Le epitope via six hydrogen (H) bonds, highlighting the importance of the Le epitope binding site in BV. In contrast, the H epitope/acetamido binding site that is commonly observed in NV, FUV and TCH is missing or greatly degenerated in BV. In fact, the H epitope of the Le^b^ and Le^y^ antigens played a minor role in the binding outcomes as it does not at all participate in binding to Le^y^, or interacting with the Le^b^ through a single H bond among the eleven H bonds in total. These data explain why BV does not bind to the A and H antigens.

The Gal binding site is presented in all the four GI NoVs whose structures of the HBGA binding interfaces are known and two of their amino acid compositions (Asp and Ser) are conserved among GI genotypes (Figs. 1 and 6). This Gal binding site was proposed as the central binding pocket (CBP) of the GI binding interfaces (Tan and Jiang 2014) due to the fact that it forms the most H bonds with the β-Gal (being designated as the major binding saccharide, MaBS) of the HBGAs based on the two known structures of GI.1 NV and GI.2 FUV (Bu et al., 2008; Choi et al., 2008; Kubota et al., 2012). The recent structural data of GI.7 TCH (Shanker et al., 2014) and GI.8 BV (this study) support this hypothesis. The occurrence of the Gal binding site is apparently independent from the binding profiles of GI NoVs. The conservation of the CBP suggests that the β-Gal functions as a strong selection factor in the evolution of the HBGA binding interfaces of GI NoVs, which is required for viral survival.

In contrast to the conserved Gal binding site, the H epitope/acetamido- and the Le epitope binding sites are apparently variable and even dispensable. The H epitope/acetamido binding site is presented in all NV, FUV and TCH that bind A, H and SeLe antigens, in which one residue (tryptophan, W) is conserved that interacts with the H epitope or the acetamido group through hydrophic interacion (Bu et al., 2008; Choi et al., 2008; Kubota et al., 2012; Shanker et al., 2014). However, this site is absent or plays only minor role in BV (see above) that does not bind the A and H antigens. Similarly, the Le epitope binding site is missing in NV that does not bind the seLe antigen. In FUV and TCH that bind the A, H, SeLe and seLe antigens, all the Gal-, H epitope/acetamido- and Le epitope-binding sites are presented. However, their Le epitope binding sites are formed by one or two amino acids. In contrast, the Le epitope binding site of BV is well developed, forming by five amino acids and interacting with the Le epitope via six H bonds. This type of binding pattern shift from the H binding to Le binding explain the ability of BV to bind both the SeLe and seLe antigens through the Le epitope binding site in combination with the β-Gal binding site (Fig. 6) with minor support of the H epitope, a scenario differing from NV, FUV and TCH that heavily rely on the H epitope/acetamido binding site (Bu et al., 2008; Choi et al., 2008; Kubota et al., 2012; Shanker et al., 2014). In summary, unlike the Gal binding site, the occurrence of the H epitope/acetamido and the Le epitope binding sites are variable depending on the binding profiles of the GI NoVs. In other words, the occurrence, maintenance and development of the H epitope/acetamido and Le epitope binding sites are under selection of the A/H and/or Le epitopes.

Structural and sequence comparisons among the four GI P domains indicate that the P-loop provides the major components of the Le epitope binding site (Figs. 1, 5 and 6). GI.1 NV has the shortest P-loop that could not reach to the Le epitope of a HBGA (Fig. 6A). In GI.2 FUV the much longer P-loop reaches the HBGA binding interface and two residues (G343′ and G245′) are involved in the formation of the Le epitope binding site (Kubota et al., 2012). However, the GI.8 BV has the longest P-loop that extends fully to the binding interface, resulting in three consecutive residues (S346′, T347′ and G348′) forming H bonds with the Le epitope (Figs. 1, 5 and 6). Interestingly, although GI.7 TCH has a short P loop similar to NV (Fig. 1), the distal end (G346′) of this P-loop moves upward to form an H bond with the Le epitope (Fig. 7C) (Shanker et al., 2014). Thus, both the length and sequence compositions of the P-loop are important in the formation of the Le epitope binding site. Another piece of evidence in supporting this hypothesis is the participation of V442 of the S-loop in the formation of the Le epitope binding site. BV has the shortest S-loop among GI NoVs, but its sequence allow a distal residue (V442) extending from the P1 subdomain to the surface of the P dimer forming an H bond with the Le epitope, a scenario that is seen for the first time. These data collectively show the flexibility of the H epitope and the Le epitope binding sites, in contrast to the conserved Gal binding site.

Another important observation of this and a previously study (Shanker et al., 2014) is the five surface loops with highly variable sequences and lengths (Fig. 1). These loops form the major structures of the top surface of the P dimers of GI NoVs (Fig. 3). As a result, the variable sequences and lengths of these loops lead to the changes of the major surface conformations among GI NoVs and, in turn, the changes of the antigenic features among the GI genotypes. Therefore, further study of the variations of these loops is necessary to understand the principle and trends of how these loops change evolutionarily, which would provide useful information for future prediction of the antigenic types. In this context, the available structural data of the four GI NoVs would provide useful models for such future studies.

The importance of the individual amino acids for the structural and functional integrity of the HBGA binding interface of BV was studied by site-directed mutagenesis. The fact that a single residue mutation wiped out the binding function completely indicated the requirement of the subtle structure of the binding interface. On the other hand, two single mutations (S346A and T347A) did not affect the binding outcomes. Crystal structure shows that S346 interacts with the Le epitope through its oxygen atom (= O) of the carboxyl group. This backbone atom is also present in alanine, which may be the reason for the unchanged binding of the S346A mutant. In addition, both threonine (T347) and alanine are tiny amino acids, sharing similar structures, although they are polar and nonpolar amino acids, respectively. Thus, a replacement of T347 with an alanine might not lead to a damage of the structural integrity and the function of the binding interface. The observed increased binding activity of the T397A mutation (Fig. 8K), might be a result of the removal of certain structure constraint due to the bigger “bump” of T397 in the wild type, which was replaced with a smaller alanine in the mutant. In fact, different replacements of amino acids in the binding interfaces of both GI and GII NoVs are often seen, which serve as examples of some amino acids are replaceable in functional HBGA binding interfaces.

In summary, we have elucidated the structures of the HBGA binding interface of the GI.8 BV that represents a unique evolutionary path selected by the host HBGAs. While the ability of binding to the β-Gal through the CBP (the Gal binding site) maintained well, the BV degenerates the H epitope/acetamido binding site that is important in the other GI NoVs and develops a Le epitope binding site differing from those of the other three GI NoVs. This type of relative conservation of the CBP and flexibility of the surrounding regions is similar to that of the GII NoVs, although GI and GII NoVs have distinct HBGA binding interfaces. Therefore, GI and GII NoVs maintain as two distinct genetic lineages characterized by their conserved CBPs. Within each genogroup further divergences in recognizing different HBGAs occur through mutations in the flexible regions surrounding the CBP under selection by hosts. Thus both the CBP and the flexible regions are important for NoVs as a successful human pathogen. While the CBP maintains the species, the variable surrounding regions enable NoVs to spread to different human populations by adaptation. This may be the scenario of huNoVs seen today.

## MATERIALS AND METHODS

### Protein cloning, expression, purification and crystallization

The cDNA fragment encoding the P domain of BV VP1 (GI.8, GeneBank accession No. AF538679.1), including amino acid sequences from Q227 to 526L, was cloned into pGEX-6P-1 expression vector (GE Healthcare Life Sciences) and was expressed in *E. coli BL21* (*DE3*). The expression and purification methods were similar to those for VA207 P protein as described previously (Chen et al., 2011). The final purified P protein was concentrated to 10 mg/mL and crystallized with hanging drop vapor diffusion method by mixing equal volume of P protein with reservoir solution containing 0.1 mol/L LiCl, 18% (*w*/*v*) PEG 3350 and 10% (*v*/*v*) 2-Methyl-2,4-Pentanediol (MPD) at 16°C. Growth of multiple crystal clusters could be detected in about a week and seeding technique was used for growth of single crystals qualified for data collection. For complex crystal growth, the P protein (14 mg/mL) was mixed with equal volume of Le^b^ [α-Fuc-(1,2)-β-Gal-(1,3)-(α-Fuc-(1,4))-GlcNAc] (Sigma, product number: L7659) or Le^y^ [α-Fuc-(1,2)-β-Gal-(1,4)-(α-Fuc-(1,3))-GlcNAc] (Sigma, product number: L7784) tetrasaccharide to a final molar ratio of 1:40, incubated for 1 h at 4°C and then crystallized with same reservoir solution. The crystallization drops were seeded the next day with crushed native protein crystals and the complex crystals could be grown within 5 days.

### Data collection and processing

Native and complex crystals were harvested, briefly soaked for 10 s in cryoprotectant composed of corresponding reservoir solution plus 15% (*v*/*v*) glycerol before mounted for diffraction test and data collection. The P protein native and Le^b^ complex data were collected at beamline 17A and 1A of Photon Factory of KEK Japan and complex Y diffraction data were collected at beamline 17U of Shanghai Synchrotron Radiation Facility (SSRF), all at the temperature of 100 K and wavelength of 1.0000 Å. X-ray diffraction data were indexed, integrated and scaled by HKL2000 (Otwinowski and Minor, 1997) software package. Statistics for data collection and processing are summarized in Table 1.

### Structure determination and analysis

The crystal structure of Norwalk virus P protein (PDB entry: 2ZL7) was used as the starting model by the program Phaser (McCoy et al., 2007) to solve the phases of the Boxer P protein structure. Due to the high resolution (~1.6 Å) of density map, we applied Autobuild from Phenix software package(Adams et al., 2002) to automatically build the main P protein structure before manually adjust the structure in COOT (Emsley and Cowtan, 2004) guided by (2*F*_o_ - *F*_c_) and (*F*_o_ - *F*_c_) electron density maps, where Fo and Fc are the observed and calculated structure factors, respectively. The programs REFMAC(Murshudov et al., 1997) and Phenix were also applied for further structure optimization before water molecules were added to the (*F*_o_ - *F*_c_) electron density map peaks (>2.5 *σ*) where they could form stable hydrogen (H) bonds (2.6–3.4 Å) with nearby amino acid residues. The final structure of P protein was validated with PROCHECK(Laskowski et al., 1993) prior to deposition to the PDB databank. The structures of P protein complexed with Le^b^ and Le^y^ tetrasaccharides were solved with the native P protein structure as search model, optimized and validated in similar ways. Analyses of final structures were performed by programs Edpdb(Zhang and Matthews, 1995) and Pymol (DeLano Scientific LLC).

### Protein data bank deposition

The coordinates, structure factors and other related information of the structures for native BV P protein (PDB entry: 4RDJ) and its complexes with Leb (PDB entry: 4RDK) and Ley (PDB entry: 4RDL) have been deposited in the Protein Data Bank, Research Collaboratory for Structural Bioinformatics, Rutgers University, New Brunswick, NJ, USA.

### Expression and purification of BV P particles with single mutations

Single amino acid mutations were introduced to the HBGA binding site of the P protein of BV through site-directed mutagenesis using the wild type construct of P domain (in plasmid pGEX-4T-1, GE Healthcare Life Sciences) as template. Site-directed mutagenesis was performed using the QuickChange Site-Directed Mutagenesis Kit (Agilent Technology, CA, USA) and corresponding primer pairs with designed mutations. The mutated P proteins were expressed and purified by *E. coli* system (BL21) as P particles as described previously (Tan et al., 2008; Tan and Jiang, 2005; Tan et al., 2009). The GST-P domain fusion proteins were digested by thrombin to release the P proteins that self-assembled into P particles. The P particle formation was determined by gel filtration chromatography using a size-exclusion column Superdex 200 (GE Healthcare Life Sciences, Piscataway, NJ) powered by an AKTA-FPLC system (model 920, GE Healthcare Life Sciences, Piscataway, NJ) followed by SDS-PAGE electrophoresis, in which the P particles form a peak at 830 kDa. The efficiency of P particle formation was ~80%. None of the designed single residue mutations in this study affected the P particle formation.

### HBGA binding assays

These were performed as described elsewhere (Huang et al., 2005). The affinity-column purified BV P particles were first diluted to 0.2 mg/mL as starting solutions. They were then diluted further in a 2-fold-series to indicated concentrations directly on the Elisa plates that had been coated with saliva samples. A panel of synthetic oligosaccharides representing types A, B, H1, H2, Le^a^, Le^b^, Le^x^, Le^y^ and three well-characterized saliva samples with known HBGA phenotypes of Le^a^, Le^b^ and Le^y^ were used for the binding assays.

### HBGA phenotyping of saliva samples

This was performed by EIA assays using the corresponding monoclonal antibodies (Mabs) against individual HBGAs as described previously (Huang et al., 2005; Tan et al., 2008). Briefly, boiled and diluted saliva samples were coated on microtiter plates. Corresponding Mabs (1:100) against individual antigens (A, B, H1, H2, Le^a^, Le^b^, Le^x^, Le^y^) (Signet Laboratories Inc., Dedham, MA) were added followed by incubation with corresponding secondary antibody horseradish peroxidase (HRP) conjugates (Immunology Consultants Laboratory Inc., Newberg, OR). The color signal was displayed by HRP substrate reagents (optEIA, BD Bioscience, San Diego, CA).

## ABBREVIATIONS

BV: Boxer virus
CBP: central binding pocket
Gal: galatose
huNoVs: human noroviruses
HBGAs: histo-blood group antigens
MaBSs: major binding saccharides
MiBSs: minor binding saccharides
VLPs: virus-like particles
P domain: protruding domain
