# Erratum to: Crystal structures of GI.8 Boxer virus P dimers in complex with HBGAs, a novel evolutionary path selected by the Lewis epitope

**DOI:** 10.1007/s13238-016-0266-5

**Published:** 2016-04-20

**Authors:** Ning Hao, Yutao Chen, Ming Xia, Ming Tan, Wu Liu, Xiaotao Guan, Xi Jiang, Xuemei Li, Zihe Rao

**Affiliations:** National Laboratory of Biomacromolecules, Institute of Biophysics, Chinese Academy of Sciences, Beijing, 100101 China; University of Chinese Academy of Sciences, Beijing, 100049 China; Division of Infectious Diseases, Cincinnati Children’s Hospital Medical Center, Cincinnati, OH 45229 USA; University of Cincinnati College of Medicine, Cincinnati, OH 45267 USA

## Erratum to: Protein Cell 2015, 6(2):101–116 DOI 10.1007/s13238-014-0126-0

In the original publication of the article, the contents in Acknowledgements section are published incomplete. The updated contents in Acknowledgements section is provided in this erratum.

### Acknowledgements

The research described in this article was supported by the National Basic Research Program (973 Program) (Nos. 2011CB910304 and 2011CB915501), the External Cooperation Program of the Chinese Academy of Sciences (Grant No. GJHZ1405), and the National Natural Science Foundation of China (Grant Nos. 31400639 and 31170702). The research of Xi Jiang and Ming Tan’s labs is supported by the US National Institute of Health (R01 AI089634/P01 HD13021 to X.J. and R21 AI092434/NCRR 8UL1TR000077-04 to M.T.).

